# The COVID-19 Community Research Partnership: a multistate surveillance platform for characterizing the epidemiology of the SARS-CoV-2 pandemic

**DOI:** 10.1093/biomethods/bpac033

**Published:** 2022-11-28

**Authors:** The COVID-19 Community Research Partnership, Thomas F Wierzba, John Walton Sanders, David Herrington, Mark A Espeland, John Williamson, Morgana Mongraw-Chaffin, Alain Bertoni, Martha A Alexander-Miller, Paola Castri, Allison Mathews, Iqra Munawar, Austin Lyles Seals, Brian Ostasiewski, Christine Ann Pittman Ballard, Metin Gurcan, Alexander Ivanov, Giselle Melendez Zapata, Marlena Westcott, Karen Blinson, Laura Blinson, Mark Mistysyn, Donna Davis, Lynda Doomy, Perrin Henderson, Alicia Jessup, Kimberly Lane, Beverly Levine, Jessica McCanless, Sharon McDaniel, Kathryn Melius, Christine O'Neill, Angelina Pack, Ritu Rathee, Scott Rushing, Jennifer Sheets, Sandra Soots, Michele Wall, Samantha Wheeler, John White, Lisa Wilkerson, Rebekah Wilson, Kenneth Wilson, Deb Burcombe, Georgia Saylor, Megan Lunn, Karina Ordonez, Ashley O'Steen, Leigh Wagner, Michael S Runyon, Lewis H McCurdy, Michael A Gibbs, Yhenneko J Taylor, Lydia Calamari, Hazel Tapp, Amina Ahmed, Michael Brennan, Lindsay Munn, Keerti L Dantuluri, Timothy Hetherington, Lauren C Lu, Connell Dunn, Melanie Hogg, Andrea Price, Marina Leonidas, Melinda Manning, Whitney Rossman, Frank X Gohs, Anna Harris, Jennifer S Priem, Pilar Tochiki, Nicole Wellinsky, Crystal Silva, Tom Ludden, Jackeline Hernandez, Kennisha Spencer, Laura McAlister, William Weintraub, Kristen Miller, Chris Washington, Allison Moses, Sarahfaye Dolman, Julissa Zelaya-Portillo, John Erkus, Joseph Blumenthal, Ronald E Romero Barrientos, Sonita Bennett, Shrenik Shah, Shrey Mathur, Christian Boxley, Paul Kolm, Ella Franklin, Naheed Ahmed, Moira Larsen, Richard Oberhelman, Joseph Keating, Patricia Kissinger, John Schieffelin, Joshua Yukich, Andrew Beron, Johanna Teigen, Karen Kotloff, Wilbur H Chen, DeAnna Friedman-Klabanoff, Andrea A Berry, Helen Powell, Lynnee Roane, Reva Datar, Colleen Reilly, Adolfo Correa, Bhagyashri Navalkele, Yuan-I Min, Alexandra Castillo, Lori Ward, Robert P Santos, Pramod Anugu, Yan Gao, Jason Green, Ramona Sandlin, Donald Moore, Lemichal Drake, Dorothy Horton, Kendra L Johnson, Michael Stover, William H Lagarde, LaMonica Daniel, Patrick D Maguire, Charin L Hanlon, Lynette McFayden, Isaura Rigo, Kelli Hines, Lindsay Smith, Monique Harris, Belinda Lissor, Vivian Cook, Maddy Eversole, Terry Herrin, Dennis Murphy, Lauren Kinney, Polly Diehl, Nicholas Abromitis, Tina St Pierre, Bill Heckman, Denise Evans, Julian March, Ben Whitlock, Wendy Moore, Sarah Arthur, Joseph Conway, Thomas R Gallaher, Mathew Johanson, Sawyer Brown, Tina Dixon, Martha Reavis, Shakira Henderson, Michael Zimmer, Danielle Oliver, Kasheta Jackson, Monica Menon, Brandon Bishop, Rachel Roeth, Robin King-Thiele, Terri S Hamrick, Abdalla Ihmeidan, Amy Hinkelman, Chika Okafor, Regina B Bray Brown, Amber Brewster, Danius Bouyi, Katrina Lamont, Kazumi Yoshinaga, Poornima Vinod, A Suman Peela, Giera Denbel, Jason Lo, Mariam Mayet-Khan, Akash Mittal, Reena Motwani, Mohamed Raafat, Evan Schultz, Aderson Joseph, Aalok Parkeh, Dhara Patel, Babar Afridi, Diane Uschner, Sharon L Edelstein, Michele Santacatterina, Greg Strylewicz, Brian Burke, Mihili Gunaratne, Meghan Turney, Shirley Qin Zhou, Ashley H Tjaden, Lida Fette, Asare Buahin, Matthew Bott, Sophia Graziani, Ashvi Soni, Guoqing Diao, Jone Renteria, Christopher Mores, Abigail Porzucek, Rebecca Laborde, Pranav Acharya, Lucy Guill, Danielle Lamphier, Anna Schaefer, William M Satterwhite, Anne McKeague, Johnathan Ward, Diana P Naranjo, Nana Darko, Kimberly Castellon, Ryan Brink, Haris Shehzad, Derek Kuprianov, Douglas McGlasson, Devin Hayes, Sierra Edwards, Stephane Daphnis, Britnee Todd, Atira Goodwin, Ruth Berkelman, Kimberly Hanson, Scott Zeger, Johns Hopkins, Cavan Reilly, Kathy Edwards, Helene Gayle, Stephen Redd

**Keywords:** COVID-19, epidemiology, surveillance, cohort, serology, symptoms, methodology, electronic health records

## Abstract

The COVID-19 Community Research Partnership (CCRP) is a multisite surveillance platform designed to characterize the epidemiology of the Severe Acute Respiratory Syndrome Coronavirus-2 (SARS-COV-2) pandemic. This article describes the CCRP study design and methodology. The CCRP includes two prospective cohorts, one with six health systems in the mid-Atlantic and southern USA, and the other with six health systems in North Carolina. With enrollment beginning in April 2020, sites invited persons within their healthcare systems as well as community members to participate in daily surveillance for symptoms of COVID-like illnesses, testing, and risk behaviors. Participants with electronic health records (EHRs) were also asked to volunteer data access. Subsets of participants, representative of the general population and including oversampling of populations of interest, were selected for repeated at-home serology testing. By October 2021, 65 739 participants (62 261 adult and 3478 pediatric) were enrolled, with 89% providing syndromic data, 74% providing EHR data, and 70% participating in one of the two serology sub-studies. An average of 62% of the participants completed a daily survey at least once a week, and 55% of the serology kits were returned. The CCRP provides rich regional epidemiologic data and the opportunity to more fully characterize the risks and sequelae of SARS-CoV-2 infection.

## Introduction

The emergence of the SARS-CoV-2 pandemic led to the urgent need for evidence-based answers to pressing clinical and public health questions. Initial efforts focused on counting and describing severe cases or monitoring clinical volumes and establishing patient registries [1]; however, these methods are limited [1–4]. While essential for public health planning, these study designs may be insufficient to answer the following essential questions:

1. What is the spectrum of SARS-CoV-2 disease in the population? The importance of asymptomatic carriers and mild cases on community spread cannot be overstated, and assessing only those needing hospital care or those presenting for testing cannot address this issue.
2. Who is at risk from SARS-CoV-2 transmission and disease? Larger studies of more diverse populations followed over time are needed to investigate disparities by race/ethnicity, healthcare worker status, age, and comorbidities.
3. What are the correlates of protection against SARS-CoV-2 infection and outcomes? Uncertainty remains about the level of protection from natural infection with varying degrees of symptom severity, and the real-world efficacy of the various vaccines, especially as new variants emerge and become prevalent.
4. What are the sequelae of SARS-CoV-2 infection at the population level? By focusing entirely on hospitalization and death, many of the impacts of SARS-CoV-2 infection in the community may be missed.

Real-world cohorts remain critical to addressing these growing research gaps. The COVID-19 Community Research Partnership (CCRP) was formed to address these limitations. The CCRP is a health systems-based surveillance platform that includes a complementary triad of regular electronic surveys, longitudinal serologic testing, and electronic health record (EHR) data designed to more accurately characterize the epidemiology of the SARS-CoV-2 pandemic. The CCRP leverages tools available through mobile technologies and at-home laboratory testing across large sample sizes at multiple sites [1] to characterize epidemiologic cohorts. In this article, we describe the study design and methodology of the CCRP.

## Study objectives

The primary objective of the CCRP was to collect more comprehensive data on the SARS-CoV-2 pandemic in the USA using a cohort approach in order to provide timely and robust evidence for clinical and public health decision-making. Specifically, the goal of the CCRP was to collect longitudinal information from four data sources: (i) daily self-reporting of symptoms, testing, and behaviors; (ii) comorbidities, medications, medical encounters, and hospitalizations documented in the EHR; (iii) SARS-CoV-2 serology and viral surveillance results from targeted subsets within the study; and (iv) intermittent participant questionnaires designed to capture supplementary data. The combination of these data sets provided an opportunity to address a diverse set of questions about the SARS-CoV-2 pandemic across the epidemic spectrum. Some key hypotheses that can be tested include: what is the clinical presentation of SARS-CoV-2 infection and how do these differ by patient characteristics (e.g. age, comorbidities, prior infection), vaccination, and time; what are the risks factors for infection and clinical disease in demographic and patient subgroups; do serological responses to prior infection whether asymptomatic or symptomatic correlate with protection; and what are the acute and chronic sequelae from infection?

## Materials and methods

### Overall cohort structure

The CCRP is composed of two prospective cohorts: one located in North Carolina (NC State Coalition) funded by the State of North Carolina through the CARES Act, and the other in the mid-Atlantic and southern USA (National Coalition) funded by the Centers for Disease Control and Prevention (CDC). Each cohort includes six sites with two sites included in both cohorts.

### Study sites

**Figure 1.**
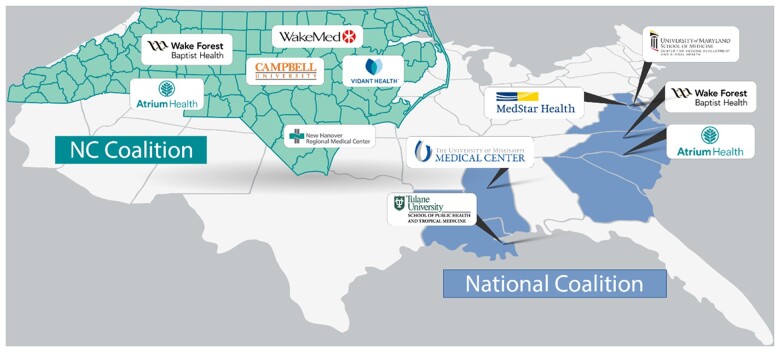
COVID-19 CCRP site locations.

The sites in the National Coalition of the CCRP include Wake Forest Baptist Health (WFBH), Atrium Health, MedStar Health/Georgetown University, the University of Maryland School of Medicine/University of Maryland Medical System, the University of Mississippi Medical Center, and Tulane University. The sites in the NC State Coalition of the CCRP include WFBH, Atrium Health WakeMed Health, New Hanover Regional Medical Center, Vidant Health, and Campbell University, WFBH and Atrium Health in both cohorts (Fig. 1). Supplementary Table S1 provides specifics of site eligibility, start dates, and recruitment that were different from that of the overall CCRP. These two cohorts were linked by common design features and data coordination but differ in serologic testing platforms and sub-study activity (described in detail below). All sites utilize a common Data Coordinating Center (DCC) at the George Washington University Biostatistics Center, a common research program management office at Vysnova Partners, Inc., and Call Centers that respond to participants’ inquiries and technical problems. Further details on the study organization are included in Supplementary Fig. S1. Data from the two cohorts were merged in a common database, with tags indicating which participants from WFBH and Atrium are included in each cohort, allowing analyses within the entire cohort or each cohort separately as required.

### Recruitment

Eligibility for the CCRP study was broad and included most adults, with the only inclusion requirements being an active email address and a cell phone with data capture capabilities. In addition to the adults recruited to participate in the two cohorts, pediatric participants were recruited to the NC Coalition. Initially, invitations to participate in the CCRP were sent out through participating healthcare systems beginning in March 2020 at WFBH and progressively included additional sites as funding allowed. Healthcare workers were oversampled. Additionally, sites had the option to invite community members outside of the participating healthcare system to participate in the CCRP. Recruitment included multiple methods: (i) email or texts using the health systems’ patient communication portals (e.g. MyChart [5]; (ii) social media and public relations campaigns led by individual sites; (iii) virtual and in-person community outreach; and (iv) a website accessible to the public with information about the CCRP (http://www.covid19communitystudy.org/index.html). See Supplementary Table S1 for additional detail about the recruitment strategy by the site.

### Daily syndromic surveillance and supplemental surveys

Following informed consent provided through secure web-based systems, participants completed a one-time enrollment questionnaire (Supplementary Fig. S2), which was immediately followed by a survey questionnaire that collected basic demographics (age, sex, and race/ethnicity), address, and if they were a current healthcare worker (Supplementary Fig. S3). Following the consent and enrollment process, participants completed daily electronic surveys via computer or smartphone using one of the two electronic systems, the Oracle Patient Monitoring System (PMS) (Oracle Corporation, Redwood Shores, California) or the SneezSafe application (Sneez, LLC, Winston Salem, NC, USA).

The daily syndromic survey asked participants to log the presence or absence of a variety of symptoms related to COVID-19 infection (Supplementay Fig. S4). Additional questions included mask use, care-seeking behaviors, known COVID-19 exposures, and self-reported SARS-CoV-2 testing results. Healthcare workers reporting COVID-19 contacts also provided data on frequency and type of personal protective equipment used at the time of potential exposure. A question about COVID-19 vaccination status and vaccine type was added in September 2020. Daily data entry typically took ∼30 s.

In addition, all CCRP participants completed periodic supplementary surveys through Oracle PMS or SneezSafe. Surveys were developed to be responsive to changing trends and newly arising research questions during the pandemic. To date, supplementary surveys have focused on risk mitigation behavior such as mask use during the Thanksgiving and winter holidays [6]; intentions and hesitancy to vaccinate; a household survey concerning family members (e.g. number, medical history, family income); and an individual adult survey exploring comorbidities, health behaviors (e.g. flu vaccination, alcohol, smoking), education, and occupation. These surveys have been and are continuing to be described in detail in relevant manuscripts.

### Electronic health record

A subset of CCRP participants, 67%, agreed to have their personal EHR data extracted and included in the study. Participants were matched to their EHR by each health system using patient-provided characteristics including name, birth date, and address, and then authenticated. To capture health conditions prevalent before the pandemic and ensure that pertinent diagnoses were not missed due to potentially reduced healthcare use during the pandemic, the EHR was abstracted from a look-back period that started in January 2018. Key EHR data are extracted at quarterly intervals to identify patient characteristics, health service utilization, diagnoses, medications, procedures, and lab test results throughout the study. The majority of EHR content is made up of readily identifiable structured data fields, extracted using formats based on the Fast Healthcare Interoperability Resources standard, and used established standards (e.g. ICD10 diagnosis codes, procedure codes, medications, patient demographics, insurance, vital signs, test results, health behaviors) where possible. Data are checked for quality to remove impossible values and harmonized across participating healthcare systems to the extent possible to enable meaningful analysis over the CCRP study population. Health conditions of interest extracted from the EHR are listed in Supplementary Table S2.

### Longitudinal serology testing

A subset of the participants from the CCRP was selected for repeated, longitudinal, at-home serologic sampling. Selected participants were eligible to receive at least six tests over the 12-month period. The selection strategy for testing and the test platform utilized differed between the National Coalition and the NC State Coalition as further described.

### National Coalition serology testing

Within the National Coalition, investigators chose serology participants from the counties in which the participating healthcare systems provide service. The counties in the catchment area of Atrium Health and WFBH were grouped into a Southeastern area, those of Mississippi University and Tulane University into a Deep South area, and those of the University of Maryland and MedStar Health/Georgetown University into a Mid-Atlantic area. The counties in those catchment areas were characterized as urban, suburban, or rural. Participants were selected for serologic testing to represent the overall demographics (sex, race/ethnicity, age) of the area based on the 2017 American Community Survey census [7]. A target bin size was determined based on the demographics of the area, and enrollment in a stratum was capped when the target bin size was reached. Additional participants were selected by oversampling subgroups considered to be at high risk for infection or complications from infection (predominantly healthcare workers and minority populations), and a small number of additional participants were selected to allow for flexibility throughout the enrollment process.

Serologic testing for the National Coalition utilized at-home collection of dried blood spots on a Whatman 5-spot DBS specimen card. LabCorp delivered a study-branded kit with instructions for collection, two lancets, and a self-addressed return envelope to participants. Once returned, LabCorp performed a EUROIMMUN enzyme-linked immunosorbent assay from the dried blood spot targeting the SARS-CoV-2 spike protein. This test has been granted Emergency Use Authorization (EUA) from the FDA for testing on venous blood [8] and was internally validated for use with dried blood spot specimens. Testing performance is described in Supplementary Table S3. This test was expected to be positive following natural infection or vaccination. For further discrimination between natural or vaccine-induced antibodies, positive tests on the EUROIMMUN assay automatically reflexed to testing for nucleocapsid antibodies utilizing the EUA-approved Roche Elecsys Anti-SARS-CoV-2 ECLIA. A natural infection would be Roche assay positive and vaccine-induced response Roche assay negative. The dried blood spot cards with unused spots have been stored in a biorepository for future ancillary studies.

### NC State Coalition serology testing

NC State Coalition serologic testing began in April 2020 before the formation of the National Coalition. The initial cadence of testing for the NC State Coalition was every other month, with additional testing added depending on the available funding. Initially, participants were mailed a simple micro-sampling device to collect a total of 40 ml blood using two volumetric absorptive swab tips (Neoteryx LLC, Torrance, CA, USA). The tips were returned by pre-addressed mail to our laboratory for further processing using one of the two lateral flow assays (LFAs) detecting IgM and/or IgG antibodies to SARS-CoV-2 (Syntron, Syntron Bio Research Inc., Carlsbad, CA, USA or Innovita, Beijing Innovita Biological Technology, China). Later, we switched to at-home serologic testing for the NC State Coalition using a LFA, the Scanwell SARS-CoV-2 IgM IgG Test (Teco Diagnostics). Scanwell Health shipped the LFAs by mail to be performed at home by participants, and the result was recorded and interpreted via an FDA-approved smartphone application developed by Scanwell Health. Interpretation by Scanwell staff involved reading a standard three-line result for IgM, IgG, and control. Test performance [9] is described in Supplemental Table S3.

### NC State Coalition virology testing

A subset of adult and pediatric participants in the NC State Coalition who consented to at-home testing completed at-home PCR-based viral surveillance. Selected adult and pediatric participants received four viral test kits and were asked to collect a sample weekly for 4 weeks. If the participant experienced persistent or worsening cough, fever, or loss of taste, they were asked to complete and return the test kit before the end of the week.

Study-branded mailers and instructional videos were developed to enable participants to conduct their own sampling at home. For each test, the participant or their caregiver collected an oral specimen of saliva by swabbing (Curative Inc., #K00023) the inside of the mouth until the swab was saturated with saliva. The swab was then placed into a capped tube (Curative Inc., #K00016) containing DNA/RNA Shield (Zymo Research, #R1100) that in turn was placed in a return mailer. The lysis buffer inactivates the SARS-CoV-2 virus immediately reducing biosafety and shipping concerns. The molecular detection assay uses RT-PCR based on the CDC EUA adapted by Curative-Korva [10]. As the regulatory environment was in a fluid state, the latest guidance on available EUA assays for SARS-CoV-2 was generally followed. This test did not differentiate SARS-CoV-2 variants.

### Study participation and retention

**Figure 2.**
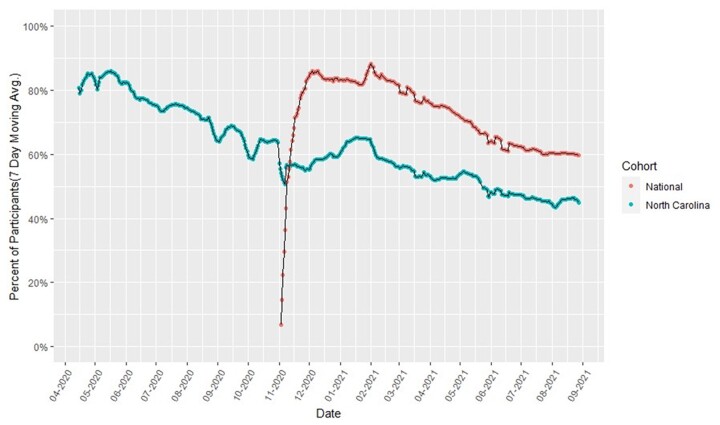
Participant retention in COVID-like illness surveillance by cohort and study date.

Although participants could join at any time, adult recruitment ended in April 2021 and pediatric recruitment ended in September 2021. By October 2021, 65 739 participants (62 261 adults and 3478 pediatric) were enrolled in the CCRP, with 89% providing syndromic data, 74% providing EHR data, and 70% participating in one of the two serology sub-studies. Accumulated enrollment in the CCRP and serology sub-studies by demographic characteristics is shown in Table 1, and demographic distribution by study site is shown in Supplementary Table S4. Retention by cohort for daily collection of syndromic data is shown in Fig. 2.

**Table 1.** Enrollment Characteristics in the CCRP and serology sub-studies

Enrollment Characteristics	Enrolled, *N* (%)	Enrolled in CDC serologic sub-study, *N* (%)	Enrolled in NC State serology sub-study, *N* (%)
Total	62 261	24 965	21 849
Age, years			
18–29	6427 (10.3)	1829 (7.3)	1872 (8.6)
30–39	11 180 (18.0)	4507 (18.1)	4058 (18.6)
40–49	11 806 (19.0)	4275 (17.1)	4796 (22.0)
50–59	12 578 (20.2)	4363 (17.5)	4991 (22.8)
60–69	12 689 (20.4)	5621 (22.5)	4456 (20.4)
70–79	6546 (10.5)	3775 (15.1)	1476 (6.8)
≥80	990 (1.6)	595 (2.4)	200 (0.9)
Sex			
Female	40 915 (65.8)	16121 (64.6)	15 974 (73.1)
Male	18 470 (29.7)	8844 (35.4)	5728 (26.2)
Not declared	2831 (4.6)	0 (0.0)	147 (0.7)
Race/Ethnicity			
White (not Hispanic/Latino)	50 485 (81.1)	20203 (80.9)	20 346 (93.1)
Black or African American	4102 (6.6)	2235 (9.0)	550 (2.5)
American Indian or Alaskan	221 (0.4)	66 (0.3)	86 (0.4)
Asian or Pacific Islander	1329 (2.1)	840 (3.4)	194 (0.9)
Hispanic or Latino	1602 (2.6)	982 (3.9)	231 (1.1)
Mixed ethnicity	918 (1.5)	574 (2.3)	163 (0.7)
Not declared/specified	3559 (5.7)	65 (0.3)	279 (1.3)
Geography/site			
WFBH	26131 (42.0)	8017 (32.1)	12 220 (55.9)
University of Maryland	5905 (9.5)	3840 (15.4)	0 (0.0)
Atrium	9557 (15.4)	3922 (15.7)	4099 (18.8)
MedStar health	12 995 (20.9)	8779 (35.2)	0 (0.0)
Tulane	244 (0.4)	187 (0.7)	0 (0.0)
University of Mississippi	478 (0.8)	220 (0.9)	0 (0.0)
Campbell	889 (1.4)	0 (0.0)	536 (2.5)
New Hanover	810 (1.3)	0 (0.0)	761 (3.5)
Vidant Health	1671 (2.7)	0 (0.0)	990 (4.5)
Wake Med	3536 (5.7)	0 (0.0)	3243 (14.8)

### Ethical considerations

The CCRP is conducted in accordance with all US federal regulations regarding the protection of human subjects. The Institutional Review Board (IRB) at WFBH serves as the central or reliance IRB. Every site IRB and the DCC have an Authorization Agreement with WFBH IRB. Any changes to protocol or implementation are notified to the site IRB for processing as needed.

A limited waiver of Health Insurance Portability and Accountability Act (HIPAA) authorization was granted by the IRB to identify potential subjects for recruitment, as allowed under 45 CFR 164.512. All data are kept confidential to the extent permitted by applicable state, local, and federal laws, and the data are stored in secured HIPAA-compliant electronic databases at George Washington University, Scanwell Health, and Oracle with password-protected access. All data are stored on secure servers in the USA. To ensure confidentiality, all data for the analysis are secured, and a coded number is used to identify participants.

Participants were provided an opportunity to consent to different aspects of the study separately, including access to their EHR, participation in symptom surveillance, serosurveillance and viral testing, and storage of residual samples. Based on EHR access, the consent was designed to serve as HIPAA authorization.

Among eligible persons or their guardians, a secure link was provided online to obtain informed consent and enrollment through one of the three electronic platforms. Sites had the option to host their own system using Research Electronic Data Capture, a software toolset and workflow methodology for electronic collection and management of data [8, 9]. An alternative consent and enrollment process used by several sites was provided by the DCC using a cybersecure, internally developed data entry, and management system known as Multimodal Integrated Data Acquisition System. A third option was provided through the SneezSafe platform, a cybersecure, phone, tablet, or computer-based application consent, and symptom-screening tool offered by Sneez, LLC. Information describing the study, common consent language, and enrollment steps were conserved regardless of the platform used. All PII was stored separately from research data, and accessible only to individuals requiring access (e.g. those responsible for mailing serology or virology test kits).

At enrollment, during informed consent, and upon receipt of a test result, participants were informed that the serology and viral diagnostic tests are for research purposes only and should not be interpreted or used for clinical diagnostic purposes. For each serology and virology result, a standardized, secure, IRB-approved message on the interpretation of a positive, negative, or equivocal result is sent to the participant, which also includes the contact information of the Call Center. Concerned volunteers were encouraged to contact the Call Center for test result information and counseling by a trained clinical professional (e.g. nurse) or, if required due to complex medical concerns, a study physician.

### Data management

A database of all study data is maintained and managed at the DCC. Data from the daily syndromic reporting and periodic supplementary questionnaires collected through SneezSafe, and all serology and virology test results, are securely loaded into the Oracle PMS. All data are downloaded three times weekly to the DCC’s secure server. EHR data are provided approximately every 3 months from each healthcare system directly to the DCC via secure file transfer protocol.

### Analysis

As this is a longitudinal study, data are appropriate for time-to-event studies such as time to infection, time to seroconversion, time to seroreversion, and time to symptom onset/clearance using parametric regression, Cox regression, Kaplan–Meier estimates, and multi-state models. Furthermore, univariate and multivariable analysis can be employed for key statistics like proportions of events and symptoms, while linear models may explore incidence rates and linear relationships between key predictors. Similarly, the likelihood of outcomes may be explored using logistic regression. The richness of this data set allows for multiple experimental design approaches beyond cohort analysis including exact and propensity score matching techniques.

When preparing for analysis, criteria for handling ambiguous responses and incomplete data varied by variable. For example, vaccination dates and doses were determined by comparing EHR results with supplemental and routine survey findings. We manually reviewed vaccination dates that differed by a few days. In some cases, individual participants were contacted for clarification. For other variables, we removed ambiguous and unconformable data (e.g. height, weight) outside of the range of physical possibility or data where participants daily reported a new diagnosis of the same disease. We deleted records for incomplete data and did not impute values for missing data.

### Data sharing and access

An internal, password-protected study website includes dynamic reports for study investigators to track recruitment, retention, and interim results. An external study website provides static reports for study participants and the public to review study demographics and results. De-identified and well-documented databases for the National Coalition are developed by the DCC and provided to the CDC quarterly. At the close of the study, the complete de-identified data will be made publicly available according to CDC policies.

The CCRP Publications and Presentations Committee (P&P) coordinates and reviews all aspects of manuscripts and presentations generated from the CCRP to prevent redundant effort, maintain consistency in reporting, and ensure scientific rigor. The P&P uses a standardized protocol for reviewing and approving manuscript proposals, conference abstracts, presentations, and penultimate drafts of manuscripts before submission to external sources. All data collected as part of the National Coalition also undergo CDC clearance.

## Discussion

As a large-scale health systems-based cohort combining daily symptom and risk behavior reporting, longitudinal serologic sampling, supplemental surveys, vaccination records, and EHR capture, the CCRP offers tremendous opportunities to obtain synergies by combining clinically relevant illness, health, and socio-behavioral data. As a longitudinal study, the CCRP also offers an opportunity to explore the changing nature of COVID-19 disease because of changes in circulating SARS-CoV-2 variants, vaccination rates, and population-level immunity. For example, investigators can calculate the proportion of antibody responses based on serology assay results and use those results to statistically model changes in population-level immunity and with that explore changes in the clinical presentation due to changing SARS-CoV-2 variants. Furthermore, investigators can calculate changes in vaccine effectiveness over time and by geographical region while considering differences based on participant clinical history, prior exposures, and demographic characteristics. The CCRP design is participant-friendly and embraces modern technologies and practical methods to overcome the limitations of more traditional studies. For these reasons, the CCRP provides a unique resource for generating evidence-based answers to questions about the SARS-CoV-2 pandemic in the USA.

The CCRP has several limitations. First, the CCRP is not a population-based study and does not consist of a random sample from the populations in the health system catchment areas, so selection bias is likely. In part, this bias is due to the study requirement of having an active email address and certain recruitment methods that relied on access to mobile technology and web or cell connectivity. CCRP participants were overrepresented by older, female, and Non-Hispanic White groups, although serology cohorts were sampled to more closely represent the demographics of surrounding counties as described above.

The decision to participate and loss to follow-up may also have been varied by demographics. As such, results may not be generalizable to broader geographical areas or to populations with less access to healthcare or electronic communications. Second, the logistics of providing serology tests to participants in their homes proved challenging, and the selection strategy for testing and the test platform utilized differed between the National Coalition and the NC State Coalition. There were clear tradeoffs between selectively reduced participation from requiring participants to have a smartphone to report the test results from one platform compared to the burden of having to take five bloodspots and return to the other test platform. Test cadence was more variable than ideal due to issues with slower test cadence in the NC State Coalition early in the study and the impact of shipping delays and slow participant response throughout the study. Nonetheless, retention in the serology cohorts was high, and this study found that at-home serology testing was generally well accepted and feasible. Third, study retention and continued participation may be impacted by known exposure, infection, and vaccination for SARS-CoV-2. Daily symptom reporting and monthly serology testing over many months can be a burden for participants. In particular, missing data may be sporadic and missing not at random. For example, a participant may stop daily symptom reporting if they do not experience any symptoms for long stretches of time but may return to daily reporting if they begin to experience symptoms. Similarly, participant engagement with serology testing may decrease after an initial positive test or vaccination. These limitations point to research that would improve CCRP and CCRP-like studies including social–behavioral investigations into outreach methods that increase enrollment of minorities and hard-to-reach populations and into technologies that improve ease of use of at-home serology testing, improve test cadence and sharing of results, and into methods to ensure the high frequency of responses and study retention.

Despite these limitations, the CCRP has several advantages over more traditional surveillance methods. The cohort study design and longitudinal data collection, including serology, allow the CCRP to assess the incidence of SARS-CoV-2 over time [11]. The serology approach from the National Coalition using a nucleocapsid confirmatory test further allowed the CCRP to assess the difference between antibody response to vaccination and natural infection. Daily symptom and self-report of SARS-CoV-2 testing with daily reminders, and a process typically taking <30 s, minimizes the limitations of typical syndromic surveillance which often suffers from misclassification due to recall bias with longer reporting intervals. By linking these data to longitudinal serology, the CCRP can characterize asymptomatic and minimally symptomatic infections, a key advantage of the CCRP design compared to that of traditional studies. Furthermore, linkage to EHR data allows for the characterization of comorbidities, healthcare encounters, clinical outcomes, and potential sequelae of infection.

The CCRP design offers several advantages in recruiting a large and diverse population. The utilization of healthcare systems as the central recruiting hubs facilitated communication through patient’s trusted healthcare systems with the potential to inspire confidence and increase enrollment and participation. Recognizing that some vulnerable populations are not well connected to healthcare systems, the CCRP included monitoring of enrollment, direct recruitment of under-represented minority groups, and over-sampling of subgroups at increased risk for infection, morbidity, and mortality (e.g. healthcare workers, minority populations, the elderly), to improve broad representation across socio-demographic groups and geographical regions. The entire study has been conducted remotely utilizing electronic informed consent, electronic/mobile technologies, and in-home sample collection and testing which enhances the involvement of participants with poor access to healthcare facilities and is supportive of social distancing measures.

## Conclusions

The CCRP is providing new and timely information about the rapidly changing SARS-CoV-2 pandemic in the USA. Symptom surveillance tied to longitudinal serology makes it possible to describe the full spectrum of disease associated with SARS-CoV-2 infection, from asymptomatic or minimally symptomatic cases to those with more severe symptoms. Serology data allow investigators to calculate the seroprevalence and incidence within demographic subgroups, and along with EHR data, to assess the risk of SARS-CoV-2 infection and severity associated with pre-existing medical conditions, and the impact of SARS-CoV-2 infection on other outcomes. Finally, the CCRP provides essential evidence of real-world vaccine efficacy and changing SARS-CoV-2 mitigation behaviors related to emerging recommendations and events. It is hoped that results from the CCRP will contribute to clinical and public health recommendations and practice to help better understand and control the SARS-CoV-2 pandemic.

## Supplementary Material

bpac033_Supplementary_Data

## Data Availability

This article describes the methods used in this study. Interested parties can refer to subsequent publications and datasets used and/or analyzed from this study. Those datasets will be available from corresponding authors upon reasonable request.
