# Supplementary figures and images for: The COVID-19 Community Research Partnership: a multistate surveillance platform for characterizing the epidemiology of the SARS-CoV-2 pandemic
Source: Biol Methods Protoc. 2022 Nov 28;7(1):bpac033. doi: 10.1093/biomethods/bpac033 (PMC9789889; doi:10.1093/biomethods/bpac033)

Supplemental Figure 4: Daily COVID-like Illness Report


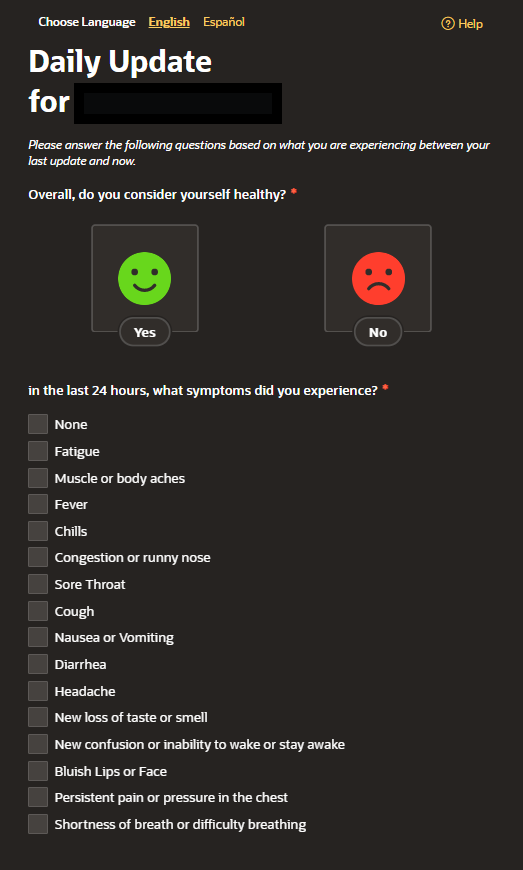


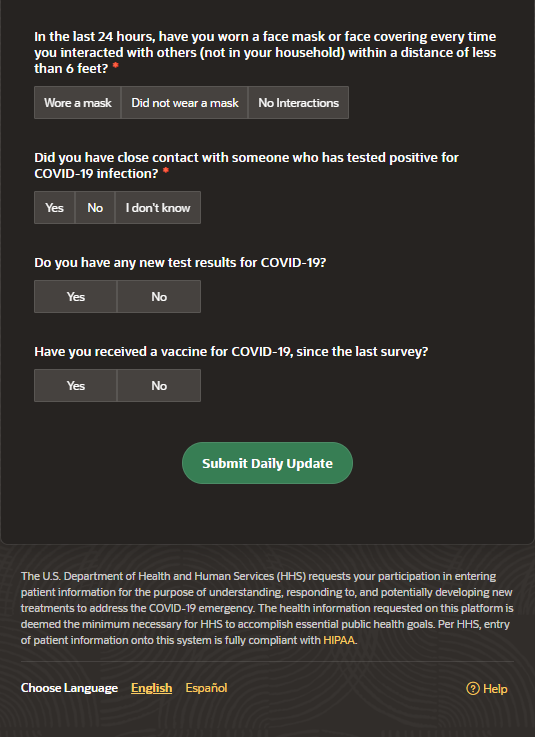

Supplement: bpac033_Supplementary_Data [file bpac033_supplementary_data.zip › Daily COVID-like Ilness Report.docx]

Supplemental Figure 2: Enrollment Questionnaire (example from Wake Forest Baptist Health)


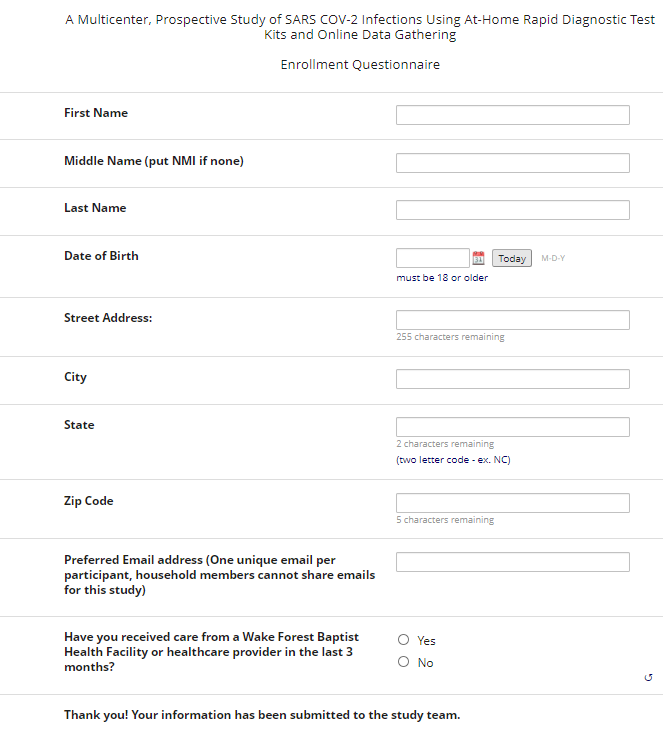

Supplement: bpac033_Supplementary_Data [file bpac033_supplementary_data.zip › Enrollment Questionnaire, Wake Forest.docx]

Supplemental Figure 1. Organizational Structure of the COVID-19 Community Research Partnership


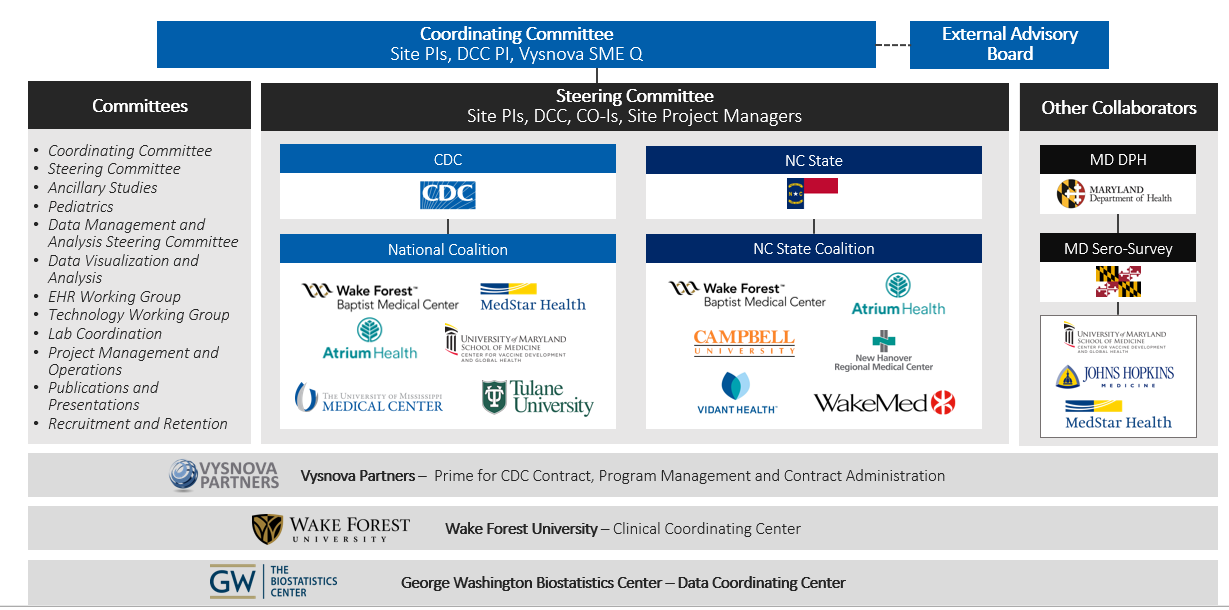

Supplement: bpac033_Supplementary_Data [file bpac033_supplementary_data.zip › Organizational Structure.docx]

Supplemental Figure 3: Registration and Demographics Form


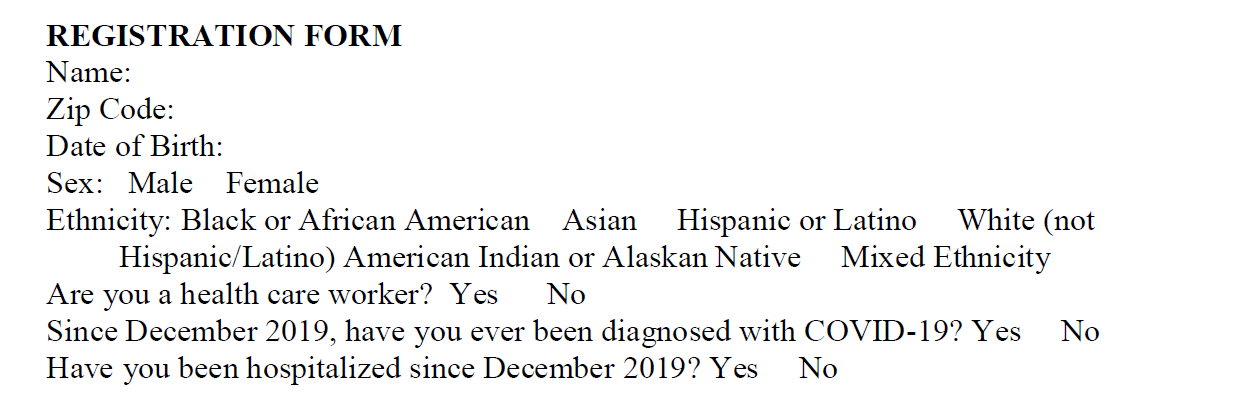

Supplement: bpac033_Supplementary_Data [file bpac033_supplementary_data.zip › Registration and Demographic Form.docx]
